# HPLC Method for Quantification of Caffeine and Its Three Major Metabolites in Human Plasma Using Fetal Bovine Serum Matrix to Evaluate Prenatal Drug Exposure

**DOI:** 10.1155/2018/2085059

**Published:** 2018-08-12

**Authors:** Rosa del Carmen Lopez-Sanchez, Victor Javier Lara-Diaz, Alejandro Aranda-Gutierrez, Jorge A. Martinez-Cardona, Jose A. Hernandez

**Affiliations:** Tecnologico de Monterrey, Escuela de Medicina y Ciencias de la Salud, Ave. Morones Prieto 3000, 64710 Monterrey, NL, Mexico

## Abstract

Caffeine is recognized as the first-line therapeutic agent for apnea of prematurity. The dosage regimen is 10 mg/kg loading dose and 2.5 mg/kg maintenance dose. However, the plasma concentration achieved, not always, is therapeutically useful. It makes necessary to increase the doses to reach plasma concentration up to 30 or 35 *μ*g/mL or even higher to attain therapeutic effect. To study why neonates have these differences, and whether these effects are linked to prenatal caffeine exposure, we had to develop an analytical method for an accurate measurement of caffeine and metabolites concentration. The analysis was carried out using fetal bovine serum (FBS) as biological matrix in a high-performance liquid chromatography with an ultraviolet detector method. This method allows acceptable chromatographic resolution between analytes in 15 minutes. It was validated and proved to be linear in the 0.1–40 *µ*g/mL range for caffeine, paraxanthine, theobromine, and theophylline in the same chromatographic analysis. Accuracy for quality control samples for intra- and interday assays was ranged from 96.5 to 105.2% and 97.1 to 106.2%. Precision had CV no more than 10% in all concentration levels for all analytes. No differences were observed between quantification in human and FBS. This method was applied to quantify plasma drug concentration in mothers and their newborns in a Mexican northeast population. In our study, we confirmed self-reported caffeine maternal intake in 85.2% (*n*=23); meanwhile, in their newborn's plasma, it was detected only in 78% (*n*=21). Caffeine plasma concentrations in mother and newborn had a linear relationship, and no differences were observed between groups (mothers versus children). These results suggest that our analytical method and substitution of biological matrix was linear, precise, and accurate for caffeine quantification and could be used for measuring prenatal exposure and let us to study, in the future, concentration differences observed during apnea clinical treatment.

## 1. Introduction

**Figure 1:**
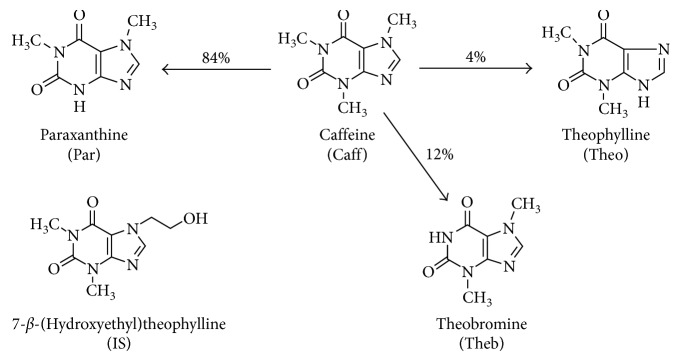
Caffeine and its major metabolites and internal standard structures.

Caffeine (1,3,7-trimethylpurine-2,6-dione) (Figure 1) is among the most consumed legal psychostimulants nowadays and is present in many and diverse kinds of foods and beverages [1, 2].

This drug has psychoactive properties. It is a slightly dissociative and stimulant drug because of its nonselective antagonist action against adenosine receptors [3, 4].

Clinically, caffeine is recognized as the first-line therapeutic agent for apnea of prematurity [5], because of its safer clinical profile compared to the older drug theophylline and its oral form aminophylline [6]. For this indication, there is an internationally proposed dosage regimen for caffeine base (10 mg/kg load dose and 2.5 mg/kg maintenance dose) [7, 8]. Effective concentrations can be found among 5 to 20 *μ*g/mL [7, 8]. In some cases, it is needed to increase plasma caffeine levels up to 30 or 35 *μ*g/mL or even higher to be able to attain therapeutic effect [7, 9]. It has been stated that there is no need to monitor caffeine concentrations due to the high therapeutic margin that it has [9], although others suggest that serum concentrations should be monitored periodically because caffeine clearance and half-life rapidly change in the postnatal period [10, 11], and under those circumstances, a method such as the one proposed here can be very useful.

The presence of multiple cytochrome P-450 isoforms (CYP450) may explain the species, strain, age, tissue, and sex differences as well as the effect of inducers, nutritional status, and human drug metabolism. In humans, caffeine is metabolized through the liver microsomal drug-metabolizing enzyme system CYP450. In neonates, the expression of this enzymatic system is dependent on their prenatal and postnatal age [10, 12]. It has been suggested that the therapeutic effects of caffeine are affected by the expression and type of CYP1A2 isoform present (main metabolic pathway), which affects not only plasma concentration of caffeine (Caf) and its metabolites (theophylline (Theo), theobromine (Theb), and paraxanthine (Par)) (Figure 1) but also its therapeutic effects [13–15].

The analytical methods used for the quantification of caffeine and its main metabolites are immunoassay with monoclonal antibodies, which presents as main disadvantage the selectivity and sensitivity of the method [16], and liquid chromatography coupled to mass spectrometry detector (LC-MS/MS), which although is a very powerful tool, but its high cost and availability for clinical application has limited its use [17, 18]. The most common analytical method used is liquid chromatography with ultraviolet detection (LC-UV) that provides adequate sensitivity and selectivity [19, 20].

Another analytical problem is to obtain caffeine-free human plasma from volunteers. Some authors have tried to use substitutes, human plasma treated with activated charcoal to remove caffeine, or the manufacture of synthetic plasma devoid of caffeine [19]. We evaluate the possibility of using bovine whole serum because it is more representative than using human plasma or serum treated with activated charcoal to deplete substances or synthetic plasma. Likewise, we consider that bovine serum is more accessible and easily freer of caffeine than that derived from a human donor [19].

Recent studies have reported that intake of caffeine during pregnancy (plasma concentration estimated by self-reported consumption) is associated with the development of sickness such as obesity and hypertension, besides others [21, 22].

Our group is interested in quantifying plasma levels of caffeine and its metabolites to determine what is the prenatal exposure to caffeine in our population and if this exposure has an impact on therapeutic response (apnea) or if it increases risk or predisposition to other diseases [21, 23].

For this reason, initially we had to develop an analytical method to quantify caffeine and its metabolites in plasma at our laboratory.

In this study, we present the results on the use of fetal bovine serum (FBS) for the development and validation of an analytical method by HPLC for the quantification of caffeine and three of its main metabolites in samples of neonates and their mothers to perform the previously mentioned studies and results of its clinical application.

## 2. Materials and Methods

### 2.1. Chemicals and Reagents

Caffeine (1,3,7-trimethylxanthine) (C0750), paraxanthine (1,7-dimethylxanthine) (D5385), theobromine (3,7-dimethylxanthine) (T4500, >98%), and 7-(*β*-hydroxyethyl)theophylline (IS) (H9006) were obtained from Sigma (St. Louis, MO, USA). Theophylline (1,3-dimethylxanthine) (J1H052) was USP standard (Rockville, MD, USA). Acetonitrile and methanol were of HPLC grade, and acetic acid was of reagent grade; all were acquired from J.T. Baker (Xalostoc, Mexico). Water used during this study was of HPLC grade (Fermont laboratories, Monterrey, Mexico). Fetal bovine serum (FBS) was used as biological matrix to prepare calibration curves and quality control samples used during method validation and quantification of newborn samples (Gibco, Life Technologies).

### 2.2. Equipment

HPLC system consisted in a quaternary pump with a degasser, and it was coupled to an autosampler and DAD–UV detector (Agilent 1200 series) (Agilent Technologies Mexico, S. de R.L. de C.V.). Separation was performed on a reverse-phase column Zorbax® SB-Aq narrow bore RR (2.1 × 100 mm, 3.5 *µ*m) (Agilent Technologies). The column oven was maintained at 40°C, while the autosampler was set at room temperature. Fifteen microliters of processed sample was injected into the HPLC system.

### 2.3. Chromatographic Conditions

For optimization of chromatographic conditions, the effects of various method parameters such as mobile phase, column, flow rate and solvent ratio, and detection system were evaluated, and the chromatographic parameters such as asymmetric factor, resolution, and column efficiency were calculated. The best results were obtained with a mixture of 10 mM phosphate buffer, pH 6.8, and acetonitrile, in a gradient phase mobile composition obtained using a gradient program (Table 1) at 0.7 mL/min. Chromatograms were recorded at 273 nm with a run time of 15 min.

At the end of each work day, the column was washed with acetonitrile : water (90 : 10 v/v) during 30 min. Chromatographic data were processed using Chemstation for LC systems software (Agilent Technologies).

### 2.4. Preparation of Stock and Working Solutions

Stock solutions of Par, Theo, and Caf (4 mg/mL) were prepared separately, dissolving an appropriate amount of each drug in diluent (Milli-Q water). Theb solution was prepared at half concentration than the others (2 mg/mL) due to theobromine having the lowest aqueous solubility compared to the rest of caffeine alkaloids. This solution was prepared adding Theb to diluent (Milli-Q water) and heating and mixing the solution prior to obtain its total volume. The solution was cooled to room temperature before it was adjusted to its final volume.

Working solutions for the different points in the calibration curve or quality control samples were prepared simultaneously for all interest drugs in water. Those solutions were obtained mixing the necessary reagent volume to attain different concentrations; 1, 3, 5, 10, 25, 50, 100, 200, 300, and 400 *µ*g/mL.

IS working solution (7-(*β*-hydroxyethyl)theophylline) was prepared at 20 *µ*g/mL in Milli-Q water. It was stored until use.

### 2.5. Standard Calibration Curves and Quality Control Samples

Seven level calibration curves were constructed by spiking water or drug-free FBS with known amounts of Caf, Theo, Par, and Theb to reach concentrations of 0.1, 0.3, 1, 2.5, 10, 20, and 40 *µ*g/mL. Three quality control samples were prepared at low, middle, and high level of the calibration curve (0.5, 5, and 30 *µ*g/mL). In all cases, the biological matrix dilution did not exceed 10%. These solutions were vortexed for one min, and then 0.5 mL aliquots were transferred into 1.5 mL Eppendorf microcentrifuge tubes and stored at −60°C until use.

### 2.6. Clinical Design

The analytical method was developed, validated, and challenged in a clinical trial. The clinical study was focused to quantify prenatal caffeine exposure, and later the analytical method will be applied to study clinical pharmacokinetics of caffeine in preterm neonates. This study reports only the results of the pilot trial of prenatal caffeine. The clinical protocol was reviewed and approved by our Institutional Research and Ethics Board registered according to Mexican law to authorize and oversee the conduction of clinical trials (13CI19039138 and CONBIOETICA-19-CEI-011-20161017). The protocol was conducted in accordance with the Declaration of Helsinki. All participants (fathers, mothers, or legal responsible) agreed to be included in the study, by signing an informed consent form.

Inclusion criteria are as follows: women 18 years old or older, pregnant with single or multiple products, gestational age less than 34 completed weeks (based on dates and confirmed with an ultrasound examination), and without a history of preeclampsia or any neonatal abnormality.

We excluded women with known fetal genetic or major malformations, very critical condition, or fetal demise.

### 2.7. Plasma Samples Collection

Blood samples (1 mL) were collected during the 15 minutes immediately after delivery from umbilical cord (neonates) or from venous puncture (mothers). Samples were collected in heparinized Vacutainer® tubes and centrifuged for 10 min at 3500 rpm under a controlled temperature of 10°C. The plasma supernatant was carefully transferred to two polypropylene tubes and frozen at −60°C until they were analyzed.

### 2.8. Samples Preparation

One hundred microliters of the IS working solution was added to 150 *µ*L of plasma samples (calibration standards, quality control samples, or mother or neonate samples) in a 1.5 mL Eppendorf tube. To this solution, we added 350 *µ*L of a 10% (v/v) acetic acid solution and 400 *µ*L of water. The tube was vortexed for 20 s and then centrifuged at 12000 rpm for 10 min at 4°C. The supernatant clear layer was collected and preprocessed in a solid-phase extraction (SPE) system.

### 2.9. SPE Cleaning

The sample pretreatment procedure was carried out on plasma by means of solid-phase extraction (SPE) on polymeric 96-well plates Strata-X™ (30 mg, 1 mL) (Phenomenex®, Torrance, CA). The plate was placed in a 96-well plate vacuum manifold system (Phenomenex, Torrance, CA). The vacuum pressure was adjusted between −15 and −20 mmHg, and each well was activated by washing with 1 mL methanol followed by 1 mL water.

One milliliter of each sample supernatant (previous step) was placed on its corresponding well and allowed to pass through low vacuum (−5 to −6 mmHg). The wells were then washed with 1 mL 5% methanol at vacuum pressure between −15 and −20 mmHg. Caffeine and its metabolites were eluted with 1 mL methanol-2% acetic acid (70 : 30) solution. The eluent was dried under N_2_ gas at 40°C. The residue was dissolved in 50 *μ*L mobile phase and filtered through 0.22 mm filters, and an aliquot (15 *μ*L) was injected into the HPLC.

### 2.10. Assay Validation

Validation was carried out following the criteria established in the Mexican regulatory guidelines [24], which are similar to the Guidance for Industry Bioanalytical Method Validation by FDA [25]. Inter- and intraday precision and accuracy were determined at low, medium, and high concentration levels on the calibration curve. Selectivity, stability, limit of quantification, and limit of detection were also evaluated [26]. Absolute extraction recoveries of Caf, metabolites, and IS were determined by comparing their respective response from quality control samples after the extraction process against their nonextracted samples above aqueous solutions. Validation results were obtained using chromatographic results and processed by SPSS software version 22.0.

### 2.11. Clinical Study Analysis

Results of caffeine and metabolites for prenatal exposure pilot study were expressed as plasma concentration for mothers and newborns. Plasma concentration differences by each mother/child pair (M/C) were calculated and used to construct a Bland–Altman plot for each compound. The differences between each M/C pair plasma concentrations were analyzed for means difference with Student's *t*-test, with a statistical significance level (*α*) of 0.05 and assuming normal data distribution. All statistical analyses were performed using SPSS software version 22.0.

## 3. Results and Discussion

### 3.1. Chromatography

We tested different analytical methods reported for caffeine and metabolite quantification [27] in order to adjust them to our laboratory conditions. However, none proved to be useful for our purposes, quantifying in a single step caffeine and its three major primary metabolites. At the beginning, we tried to develop an analytical method by fluorescence detection in an Acquity™ ultra-performance liquid chromatography (UPLC; Waters Corp., Milford, MA, USA), but it did not have enough sensitivity.

It is well known that fluorescence is more sensitive than UV for analysis of various compounds. However, fluorescence sensitivity to detect caffeine in biological samples remains a challenge.

At the beginning of our experimental process, we found a report that described fluorescence parameters for caffeine analysis. We obtained the same values of fluorescence spectrum by scanning a caffeine aqueous solution at 40 *µ*g/mL, and excitation wavelength (*λ*exc) and emission wavelength (*λ*emis) were 272 nm and 385 nm, respectively (data not shown). Recently, Weldegebreal et al. reported fluorescence caffeine wavelengths similar to those we found [28]. However, in our experiments, at the highest concentration of caffeine in the calibration curve, the signal recorded was too low. This is similar to what was reported by Weldegebreal at a greater concentration [28].

We believe that another problem could be related with the caffeine fluorescence in biological samples. Some researchers have described that caffeine and its metabolites (theophylline and theobromine) can quench fluorescence signals for proteins, amino acids, and hemoglobin [29]. But, could those analytes (protein, aromatic amino acids, and hemoglobin) quench caffeine fluorescence? Currently, we do not have data regarding this, but we believe it is possible because the static quenching mechanism is based on the noncovalent interaction characteristic of a *π*-stacked complexes [29].

Recently, a “light traffic” detector has been developed to improve caffeine fluorescence detection. This device uses a compound called “caffeine orange” to conjugate caffeine and increase about 250-fold the fluorescence signal. However, the main setback is that it is not selective and can increase fluorescence for caffeine, theobromine, theophylline, and other methylxanthines. This result suggests that fluorescence intensity signal by caffeine is too low for direct detection and needs derivatization to increase response [30].

**Figure 2:**
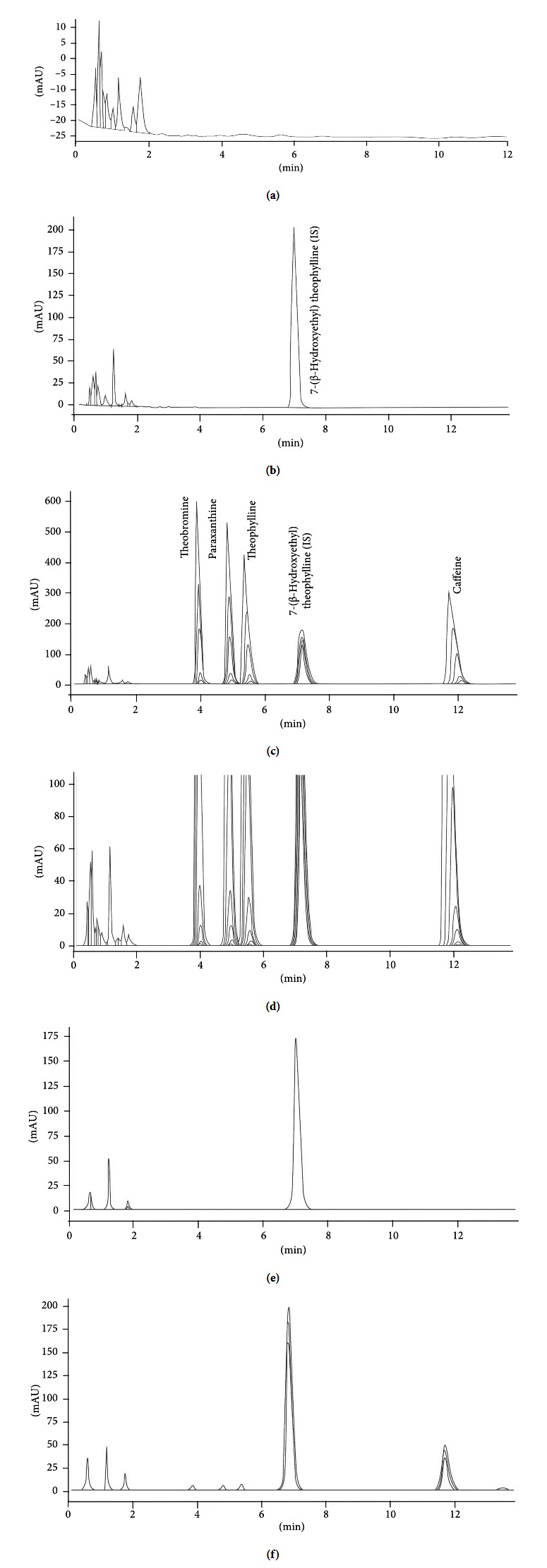
Representative chromatograms of FBS blank (a), FBS + IS (b), FBS spiked with caffeine and metabolites at all calibration curve levels lower (0.1 *μ*g/mL) and upper (40.0 *μ*g/mL) (c), and zoomed-in view showing the lower limit of quantification (d); plasma obtained from a mother volunteer and her child (e) without caffeine and metabolites (volunteer 3); and plasma obtained from a mother volunteer and a pair of her twins T1 and T2 (e) containing caffeine and metabolites (volunteer 5). Theb: theobromine (RT 3.8 min), Par: paraxanthine (RT 4.8 min), Theo: theophylline (RT 5.5 min), IS: 7-*β*-(hydroxyethyl)theophylline (RT 7.0 min), and caffeine (RT 12 min). RT: retention time.

Conversely, the HPLC/UV method had enough sensitivity for 0.1 *μ*g/ml for each analyte in solution. We tested C8 and C18 columns with different pH values or mobile phase composition. However, retention time for each analyte was too short (losing resolution) or too long (increasing analytical run time). Our analytical method had an excellent chromatographic behavior. We were able to separate Caf, Theo, Theb, Par, and IS in 15 minutes using a chromatographic column designed to retain hydrophilic compounds using highly aqueous mobile phase without “phase collapse.” This method was faster than other HPLC-UV methods that have been reported previously with a running time higher than 20 min or even up to 60 min [31]. Caf, metabolites, and IS had a continuous resolution factor of about 1.2 between contiguous peaks. Retention times were 3.8, 4.8, 5.5, 7.0, and 12.0 min for Theb, Par, Theo, internal standard (IS), and Caf, respectively.  Figure 2 shows representative chromatograms of FBS blank (Figure 2(a)); FBS + IS (Figure 2(b)); FBS spiked with caffeine and metabolites at all calibration curve levels lower (0.1 *µ*g/mL) and upper (40.0 *µ*g/mL) (Figure 2(c)) and zoom to show the lower limit of quantification (Figure 2(d)); plasma obtained from a mother volunteer and her child (Figure 2(e)) without caffeine and metabolites (volunteer 3); and plasma obtained from a mother volunteer and a pair of her twins T1 and T2 (Figure 2) containing caffeine and metabolites (volunteer 5). In all conditions, chromatographic traces were completely clean and none exhibited any type of interference throughout the entire running time.

### 3.2. Linearity and Lower Limit of Quantification (LLOQ)

**Figure 3:**
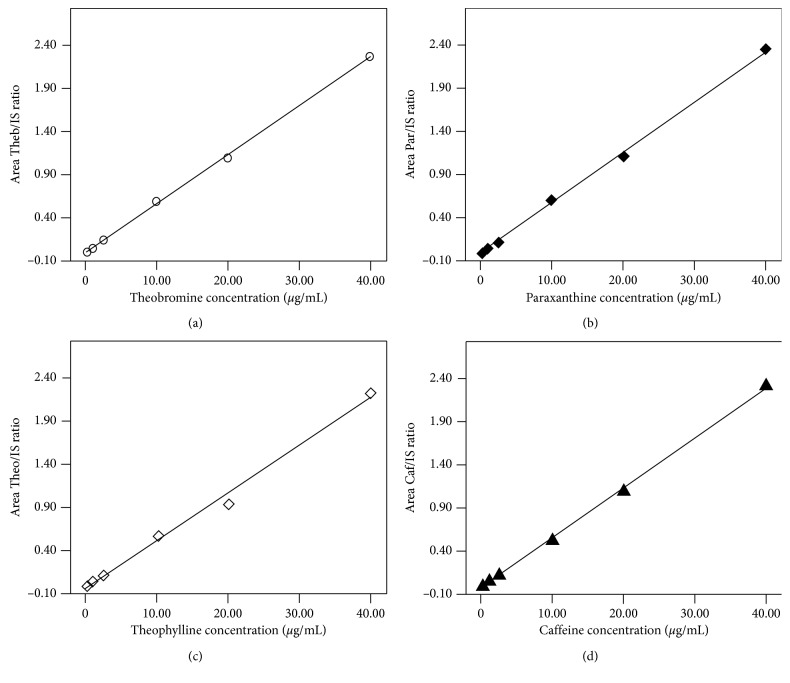
Typical calibration curves for quantification of theobromine (a), paraxanthine (b), theophylline (c), and caffeine (d) in human plasma. Theobromine (-○-), paraxanthine (-♦-), theophylline (-◊-), and caffeine (-▲-).

The calibration curves were linear over the concentration range of 0.1–40 *µ*g/mL, in a first-order model for all analytes. The linear equation for each assayed analyte was as follows: for theobromine (Theb/IS) = −0.001 + 0.056 (concentration), *r*^2^ = 0.998 (Figure 3(a)); for paraxanthine (Par/IS) = 0.006 + 0.056 (concentration), *r*^2^ = 0.997 (Figure 3(b)); for theophylline (Theo/IS) = 0.015 + 0.055 (concentration), *r*^2^ = 0.983 (Figure 3(c)); and for caffeine (Caf/IS) = −0.017 + 0.055 (concentration), *r*^2^ = 0.995 (Figure 3(d)).

For all analytes, LLOQ in BFS was 0.1 *µ*g/mL. The LLOQ had a signal-to-noise ratio higher than 10 : 1 (CV < 15%) (Figure 2(d)).

### 3.3. Recovery, Accuracy, and Precision

The optimal extraction recovery was obtained with SPE cartridges as it was described previously. To calculate absolute recovery of caffeine, metabolites, and IS, 5 sets of samples (0.5, 5, and 30 *µ*g/mL) were prepared in FBS, in human plasma, or in mobile phase, and peak areas were compared. Mean analytical recovery for all concentration levels of Theb, Par, Theo, and Caf was 92, 96, 95, and 102%, respectively, and 95% for the IS. Inter- and intraday accuracy and precision values of the assay are presented in Table 2.

No differences were observed between relative recovery of caffeine and its metabolites from FBS samples compared with human plasma assessed by Bland–Altman analysis and Student's *t*-test (Table 3). This result indicates that the method in FBS was reliable within the studied concentration range and its application to human samples analysis.

### 3.4. Stability Assays and Selectivity

Caf, Theo, Theb, Par, and IS proved to be stable in biological samples for at least 6 months at −60°C (final mean recovery of 101.7, CV 5.7%); samples were also stable for at least two freeze and thaw cycles (98%; CV 4.3%) and for at least 8 h on the worktable at room temperature (25°C; 96.5%; CV 4.5%). This was enough time according to our preparation sample method. Analytes were also stable for at least 13 h in the autosampler (101.5%; CV 6.0%), which made it possible to analyze about 50 samples in a row.

There were no endogenous compounds that interfered either with caffeine or its metabolites or with IS, even in hemolyzed or jaundiced samples. Other results of selectivity evaluation demonstrated that there were no interfering peaks from any of the following drugs: acetaminophen, ibuprofen, erythromycin, furosemide, or heparin, which are commonly used during neonatal pharmacotherapy.

### 3.5. Prenatal Caffeine Exposure

The trial was conducted from July 2013 through December 2013 in three medical centers located in Northeastern Mexico: the Hospital Regional Materno Infantil (Center 1), the Hospital Metropolitano Dr. Bernardo Sepúlveda (Center 2), and the Hospital Zambrano-Hellion (Center 3). The first two are public hospitals that harbor level II-III neonatal intensive care units and belong to the Servicios de Salud Network in Nuevo León, Mexico. The last is a private hospital with a level III neonatal intensive care unit and belongs to the academic and research health branch of Tecnológico de Monterrey in Monterrey, Nuevo León, Mexico. Seventy-three pregnant females that were admitted to the obstetric ward during labor were eligible to participate. Forty-six were excluded for different reasons: fetal death (19), malformation (1), or transfer to another unit (4), and 22 eligible mothers who declined the invitation to participate. Thus, only 27 pregnant volunteers participated in this study.

**Figure 4:**
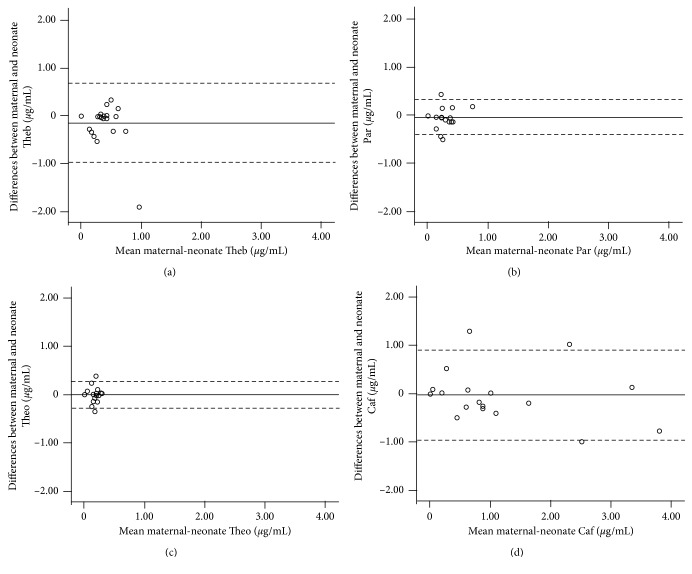
Bland-Altman plot for theobromine (a), paraxanthine (b), theophylline (c), and caffeine (d) plasma concentrations for mother versus newborn samples. Differences between mother-child pairs. Continuous line represents mean of difference between maternal concentration and neonatal concentration for each analyte. Dotted lines represent lower and upper limits of 95% confidence interval. Theb: Theobromine, Par: paraxanthine, Theo: theophylline, and Caf: caffeine.

In our study, we were able to demonstrate the maternal consumption of caffeine in 85.2% of the volunteers (*n*=23); meanwhile, in newborns' plasma, caffeine was only quantified in 78% (*n*=21). Means of plasma Caf concentration were 0.87 ± 0.22 and 0.89 ± 0.24 *µ*g/mL in mother and newborn groups, respectively. No differences were observed in occurrence and level of caffeine from other populations [32]; however, it is necessary to increase sample size to establish better conclusions. No differences were detected between groups or each pair of mother/child samples evaluated by Bland–Altman plot (Table 4, Figure 4(d)). Likewise, means of plasma concentration for Theb, Par, and Theo were 0.27 ± 0.05 and 0.42 ± 0.08 *µ*g/mL, 0.17 ± 0.05 and 0.21 ± 0.04 *µ*g/mL, and 0.12 ± 0.03 and 0.12 ± 0.03 *µ*g/mL for mother and newborn groups, respectively. No differences were detected between groups assessed by *t*-test or Bland–Altman plot (Table 4, Figures 4(a)–4(c)).

**Figure 5:**
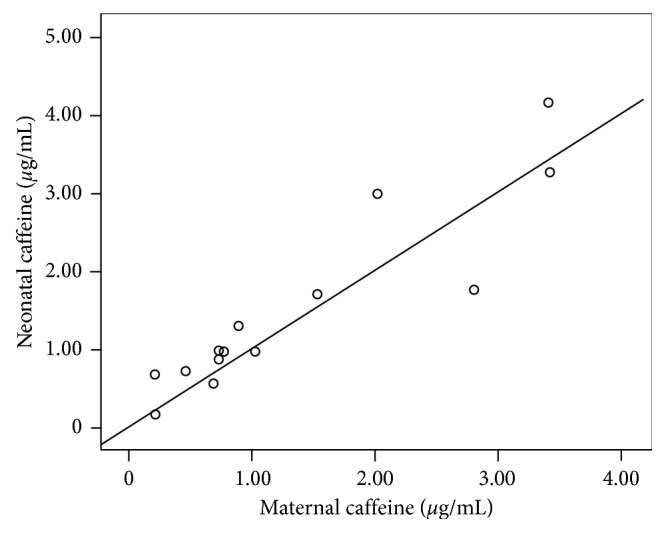
Linear regression between plasma caffeine concentrations in mother versus newborn samples.

For Caf in maternal and newborn plasma concentration, there was a positive linear regression (Figure 5). However, for Theb, Par, and Theo, the same relationship was not found. An explanation could be that those metabolites had a different transplacental transfer rate perhaps related to gestational age [33] or changes in the metabolic pathway rate for caffeine and metabolites, also in relation to gestational age as it has been reported for other drugs [34]. We need to perform a deeper study to explain differences between maternal caffeine and its metabolite concentrations and their corresponding concentrations in newborns.

## 4. Conclusion

We developed a rapid and sensitive analytical method for the simultaneous determination of caffeine and its three principal metabolites in adult and newborn human plasma using HPLC technique after solid-phase extraction with Strata-X™ plates. This method had an acceptable accuracy (96.5–105.2% for intraday and 97.1–106.2% for interday) and precision with a CV less than 10% in all concentration levels used as quality control samples for all analytes. This method is suitable for use in individuals in which blood extraction volumes are limited (150 *μ*L of plasma) due to their body size, as is the case in newborns, and it can be used for repeated sample procurement such as in pharmacokinetics studies as well as in therapeutic drug monitoring of caffeine and its metabolites in human neonates and perhaps also in small mammals (biological models).

## Figures and Tables

**Table 1:** Final gradient program for HPLC sample analysis.

Time (min)	*A* (%)	*B* (%)	Flow rate (ml/min)
0	97	3	0.7
8.5	97	3	0.7
12	92	8	0.7
13	97	3	0.7

Mobile phase A: 10 mM phosphate buffer, pH 6.8. Mobile phase B: acetonitrile 100%.

**Table 2:** Results of intra- and interday variability during validation of the HPLC method. Accuracy is expressed as percentage of the nominal value. Precision is expressed in terms of the percentage of the coefficient of variation.

Concentration (*µ*g/mL)	Interday, *n*=9, accuracy (%)/precision (CV)				Intraday, *n*=5, accuracy (%)/precision (CV)			
	Theb	Par	Theo	Caf	Theb	Par	Theo	Caf
0.5	103.7 (8.8)	96.6 (4.0)	96.5 (2.3)	100.73 (8.0)	101.2 (8.7)	97.1 (3.5)	97.5 (4.1)	98.4 (6.3)
5	100.9 (5.7)	103.2 (4.9)	99.0 (4.6)	102.2 (6.6)	100.6 (6.8)	102.1 (5.0)	99.9 (5.0)	103.4 (5.6)
30	101.9 (7.5)	102.1 (6.4)	103.2 (5.5)	105.2 (5.7)	102.9 (8.7)	101.0 (5.5)	103.0 (5.4)	106.2 (4.8)

Interday results are the accumulation of determination of 3 quality control samples in three different days for each concentration level.

**Table 3:** Differences between concentrations of caffeine and metabolites in human plasma or fetal bovine serum spiked sample.

Concentration (*µ*g/mL)	Human plasma mean (CV)			Fetal bovine serum mean (CV)			*p* value
	0.5	5.0	30.0	0.5	5.0	30.0	
Theb	0.50 (3.4)	5.21 (3.4)	31.49 (4.1)	0.52 (8.8)	5.04 (5.7)	30.57 (7.5)	NS
Par	0.50 (2.6)	5.01 (4.4)	30.92 (7.4)	0.48 (4.0)	5.16 (4.9)	30.62 (6.3)	NS
Theo	0.49 (4.2)	4.98 (4.6)	31.10 (5.2)	0.48 (2.3)	4.95 (4.6)	30.95 (5.5)	NS
Caf	0.52 (6.7)	5.03 (7.0)	32.28 (4.9)	0.50 (8.0)	5.11 (6.6)	31.57 (5.7)	NS

NS: no differences besides groups for each analyte at the same concentration.

**Table 4:** *t*-Test analysis for mean comparison between maternal and neonatal caffeine and metabolite concentration. Statistical analysis was done by comparing difference of concentrations mother-child versus zero difference.

Analyte	Mean difference	SE	95% CI difference	Diff (mother-neonate) versus zero	
Theobromine	−0.1462	0.087	(−0.327 to 0.035)	*p*=0.109	NS
Paraxanthine	−0.0352	0.037	(−0.113 to 0.042)	*p*=0.361	NS
Theophylline	−0.0044	0.028	(−0.064 to 0.055)	*p*=0.880	NS
Caffeine	−0.0194	0.097	(−0.221 to 0.182)	*p*=0.844	NS

Caffeine and its metabolites were measured by our HPLC method; CI: confidence interval; Diff: difference; SE: standard error.
